# Treatment for Apraxia of Speech in Nonfluent Variant Primary Progressive Aphasia

**DOI:** 10.3233/BEN-2012-120260

**Published:** 2013

**Authors:** M. L. Henry, M. V. Meese, S. Truong, M. C. Babiak, B. L. Miller, M. L. Gorno-Tempini

**Affiliations:** ^1^Memory and Aging CenterDepartment of NeurologyUniversity of CaliforniaSan FranciscoCAUSA; ^2^Alta Bates Medical CenterEl CerritoCAUSA; ^3^San Francisco State UniversitySan FranciscoCAUSA

**Keywords:** Apraxia of speech, treatment, speech therapy, nonfluent PPA, primary progressive aphasia

## Abstract

There is a growing body of literature examining the utility of behavioral treatment in primary progressive aphasia (PPA). There are, however, no studies exploring treatment approaches to improve speech production in individuals with apraxia of speech (AOS) associated with the nonfluent variant of PPA. The purpose of this study was to examine a novel approach to treatment of AOS in nonfluent PPA. We implemented a treatment method using structured oral reading as a tool for improving production of multisyllabic words in an individual with mild AOS and nonfluent variant PPA. Our participant showed a reduction in speech errors during reading of novel text that was maintained at one year post-treatment. Generalization of improved speech production was observed on repetition of words and sentences and the participant showed stability of speech production over time in connected speech. Results suggest that oral reading treatment may offer an efficient and effective means of addressing multisyllabic word production in AOS associated with nonfluent PPA, with lasting and generalized treatment effects.

## 1. Introduction

Primary progressive aphasia (PPA) is a degenerative neurological condition in which speech and language functions are disrupted, while other cognitive abilities remain relatively spared. The disorder results from atrophy in regions of the brain that sub-serve language. Three clinical variants of PPA are now widely accepted. These include a semantic variant, with verbal and nonverbal semantic deficits, a logopenic variant, with anomia and phonological working memory problems, and a nonfluent/agrammatic variant, with agrammatism and/or apraxia of speech [1,2].

PPA has been linked to several types of neuropathology, including tau or ubiquitin/TDP-43 positive frontotemporal lobar degeneration and Alzheimer's Disease [3,4]. While the prevalence of PPA is not known, frontotemporal dementia, which may manifest in PPA, is now recognized as a common cause of dementia affecting individuals under the age of 65 [5,6].

Relative to individuals with aphasia resulting from stroke, individuals with PPA are under-referred for speech-language pathology services [7]. Further, they are less likely to be offered behavioral treatment, in part due to lack of understanding of the disorder by referring and treating clinicians, and also due to negative assumptions regarding the feasibility and utility of behavioral treatment in patients with neurodegenerative disease. Treatment research focused on individuals with PPA has addressed restitutive treatments designed to rehabilitate impaired speech-language processes, augmentative/alternative approaches to treatment, and interventions designed to address activity and participation limitations (for reviews, see [8,9]).

Word-finding deficits (for a review, see [8]) as well as sentence production [10] and written language impairments [11] have been the object of restitutive treatments. At present, there is modest evidence supporting the utility of speech-language treatment in PPA. Re-

ISSN 0953-4180/13/$27.50 © 2013 – IOS Press and the authors. All rights reserved search reports have shown gains on language measures, which were largely restricted to treated language domains and certain treatment approaches appear to slow the progression of language deficits for specifically-trained behaviors [12]. Much remains to be learned, however, with regard to the treatment paradigms that may be beneficial for the clinical variants and stages of PPA. In addition, the long-term benefits of treatment in this population have yet to be elucidated.

The majority of research on speech-language treatment in PPA has involved individuals with semantic variant. The logopenic and nonfluent variants have received less attention. Treatment studies addressing nonfluent variant PPA have focused on linguistic deficits associated with the disorder, including verb and sentence production [13], phonological processing [14], and lexical retrieval [15]. The motor speech deficits, or more specifically, apraxia of speech (AOS), observed in nonfluent variant have not, to our knowledge, been examined with systematic treatment research. This is unfortunate, given that a gradual decline in motor planning ability for speech, or AOS, can be the most prominent and debilitating feature of nonfluent variant PPA [16].

A 2006 review of the AOS treatment literature by Wambaugh and colleagues [17] revealed that the vast majority of published AOS treatment cases were stroke patients and most of those were classified as severely apraxic. There are relatively few treatment studies examining the utility of treatment in mildly apraxic patients, perhaps because these individuals are largely intelligible and have fairly functional spoken communication. In contrast to many stroke patients with AOS, motor speech deficits in nonfluent variant are often quite mild during initial stages of the disease, and sometimes are the only deficit for a number of years. Thus, treatment approaches designed for individuals with AOS due to stroke, who are often profoundly apraxic, may not be appropriate for AOS in the context of nonfluent variant PPA. However, in PPA, AOS can be expected to worsen over time and early intervention, when the disorder is mild, may be beneficial in slowing the progression of deficits and/or providing strategies for communication in the face of a worsening impairment of motor programming for speech.

In this study, we attempted to remediate speech production for multisyllabic words via structured oral reading of text. It is a well-documented feature of AOS that production breaks down with words of increasing length and articulatory complexity [18]. Multisyllabic word production was our patient's primary difficulty and therefore, this was the level at which treatment efforts were directed. Oral text reading has been used therapeutically in individuals with aphasia with or without apraxia of speech, with benefits observed in written and spoken language [19,20]. Written word forms have also been incorporated as a supplement to other treatment approaches in acquired AOS, and have been described as a means of "intersystemic facilitation" wherein a relatively intact system or modality is used to facilitate improvement in an impaired system or modality [16]. In the case of oral reading to promote speech production, relatively preserved visual processing of orthography and conversion between orthography and phonology prior to motor implementation may serve to facilitate motor planning and execution for speech. In effect, orthography may serve to cue speech sound production by serving as a visual guide to the correct sequence of phonemes and syllables.

To our knowledge, there are no treatment studies examining oral reading as a primary means of rehabilitating speech production in AOS. In mild AOS, where production of multisyllabic words may be the most pronounced difficulty, oral reading can provide a context within which to elicit production of these words, which may not occur frequently in spontaneous speech. Oral reading also allows for rehearsal of multisyllabic word production in the context of connected speech rather than in isolation. Here we describe a single-subject treatment study wherein we implemented a novel treatment approach for a case of mild AOS in the nonfluent variant of PPA. Goals of the study were a) to determine whether structured oral reading practice can improve production of multisyllabic words read aloud, b) to examine generalization to untrained speech behaviors and contexts, and c) to assess long-term effects of training on oral reading and speech production.

## 2. Methods

### *2.1. Participant*

The participant, GA (fictitious initials), was a 73 year-old right-handed woman. She spoke English as her second (after German) but primary language. GA studied English in school and began using it as her primary language 52 years prior to this study. She obtained 20 years of education and worked for an insurance brokerage before retiring at age 55. GA had no history of developmental disorders of speech and language. At the start of treatment, she had a five-year history of progressive impairment of speech production.

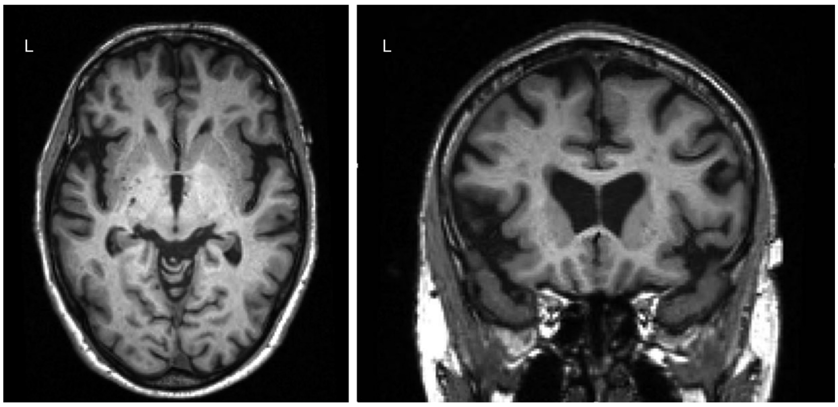

Fig. 1. Structural MRI scan obtained three months prior to initiation of treatment.

She participated in yearly research evaluations at the Memory and Aging Center (MAC) at UCSF for three years prior to her enrollment in the treatment study reported here. A consensus diagnosis of nonfluent variant primary progressive aphasia was reached by a team of clinicians (neurologist, neuropsychologist, and speech-language pathologist) at her initial visit according to published clinical criteria [1,21]. A structural MRI scan was obtained three months prior to initiation of treatment (Fig. 1). The scan revealed frontal/insular as well as temporoparietal atrophy in the left hemisphere. At the time treatment was initiated, the patient's major complaint concerned difficulty with production of multisyllabic words and an overall slowness and effortfulness of speech production, which she observed to be worsening gradually over time.

### 2.1.1. *Pre-treatment speech and language evaluation*

Pre-treatment speech and language assessment showed relatively fluent, grammatical spoken language (Table 1). The Motor Speech Evaluation [22] revealed that speech production was slow in rate with vowel and consonant distortions; sound insertions, deletions, and transpositions; pauses between words and syllables; increasing errors with increasing word length; and reduced prosodic variation. This pattern is consistent with an impairment of motor planning for speech production, or AOS [16,17,23]. Dysarthria was not noted. Syntax comprehension and production were examined using in-house assessments (for details, see [24]). On the production task, which involves generation of sentences in response to pictured scenes, as well as the comprehension task, which requires matching an auditorily-presented sentence to one of two pictures, GA performed at ceiling. Semantic processing, as measured by the picture version of the Pyramids and Palm Trees Test [25] and the Boston Naming Test [26], was spared. Reading and spelling of single words and nonwords on the Arizona Battery of Reading and Spelling (ABRS) [27] was normal.

Repetition was tested at the word and sentence level in order to assess motor speech at increasing levels of difficulty. Word repetition was examined with a list of one- and three-syllable words used previously to test speech production ability in stroke patients [28]. Words were presented auditorily and repeated five times in succession by GA. Repeated word production was spared for monosyllables, but was impaired for trisyllabic words. On a sentence repetition task adapted from Bayles [29], repetition of short sentences was near ceiling, whereas repetition of longer sentences was impaired.

Oral reading of text was examined using passages 10–14 (most difficult passages) of the Gray Oral Reading Test (GORT-4). Pre-treatment assessment of oral reading confirmed difficulty with production of multisyllabic words in sentence context. In fact, 95% of speech errors occurred on multisyllabic words, which GA typically attempted to self-correct. There was a significant proportion (49%) of repeated attempts at multisyllabic word production which were ultimately unsuccessful.

## 2.2. *Treatment design and treatment probes*

Treatment consisted of structured oral reading of text, with the goal of providing a framework for systematic rehearsal of multisyllabic word production in sentence context. During treatment sessions, GA was trained in self-detection and correction of speech errors while reading text aloud. Weekly treatment sessions were supplemented by oral reading practice at home. The treatment approach involved the following steps:

Table 1
Pre- and post-treatment assessments

| | Pre-Tx | Post-Tx | 3 mos. post | 6 mos. post | 1 year post |
|---|---|---|---|---|---|
| **Mini Mental State Exam (30)** | 29 | 26 | 29 | 29 | 30 |
| **Western Aphasia Battery** | | | | | |
| Information content (10) | 10 | 10 | 10 | 10 | 10 |
| Fluency (10) | 9 | 9 | 9 | 9 | 6 |
| Comprehension (10) | 9.75 | 10 | 10 | 9.8 | 9.75 |
| Repetition (10) | 9.4 | 9.4 | 9.2 | 9.5 | 8.9 |
| Naming (10) | 9.4 | 9.4 | 9.8 | 9.8 | 10 |
| Aphasia Quotient | 95.1 | 95.6 | 96 | 96.2 | 89.3 |
| **Motor Speech Evaluation- apraxia rating (0–7)** | 1 | 1 | 2 | 2 | 3 |
| **Motor Speech Evaluation- dysarthria rating (0–7)** | 0 | 0 | 1 | 1 | 1 |
| **UCSF sentence production (16)** | 16 | 16 | 16 | 16 | 16 |
| **UCSF sentence comprehension (24 or 48)** | 48 | 24/24 | 24/24 | 45/48 | 45/48 |
| **Pyramids and Palm Trees Test- pictures (52)** | 52 | 52 | | | |
| **Boston Naming Test (60)** | 51 | 53 | | | |
| **Boston Naming Test (15)** | | | 15 | 15 | 15 |
| **Reading words** | | | | | |
| Regular high frequency (20 or 10) | 20 | 20 | 10 /10 | 10/10 | 10/10 |
| Regular low frequency (20 or 10) | 20 | 20 | 10/10 | 10/10 | 10/10 |
| Irregular high frequency (20 or 10) | 20 | 20 | 10/10 | 10/10 | 10/10 |
| Irregular low frequency (20 or 10) | 19 | 20 | 9/10 | 10/10 | 9/10 |
| Nonwords (20 or 10) | 20 | 20 | 10/10 | 10/10 | 10/10 |
| **Spelling words** | | | | | |
| Regular high frequency (20 or 10) | 20 | 20 | 10/10 | 10/10 | 10/10 |
| Regular low frequency (20 or 10) | 20 | 20 | 10/10 | 10/10 | 10/10 |
| Irregular high frequency (20 or 10) | 20 | 19 | 10/10 | 10/10 | 10/10 |
| Irregular low frequency (20 or 10) | 20 | 18 | 10/10 | 10/10 | 10/10 |
| Nonwords (20 or 10) | 20 | 19 | 10/10 | 10/10 | 10/10 |
| **Word repetition x5 (correct repetition attempts)** | | | | | |
| Monosyllabic (120 or 60) | 120 | 120 | 60/60 | 60/60 | 60/60 |
| Trisyllabic (120 or 60) | 96 | 117 | 57/60 | 59/60 | 59/60 |
| **Sentence repetition (syllables correctly repeated)** | | | | | |
| Short sentences (60) | 59 | 60 | 60 | 60 | |
| Long sentences (135) | 106 | 121 | 116 | 111 | |

Note that shortened versions of some assessments were used at follow-up testing.

1. Text was read aloud until GA produced a word incorrectly. Incorrect production was specified as inability to produce a word in its entirety or a production with one or more "major" speech sound errors. We defined major errors as follows: sound deletion, insertion, transposition, substitution, or distorted substitution/insertion.
2. If the target was a multisyllabic word, it was underlined in the text and lines were drawn dividing the word into constituent syllables. The word was then produced syllable-by-syllable until each syllable was produced correctly, at which point the whole word was repeated in a non-syllabified manner. Single-syllable words were simply repeated (typically once) until produced correctly in isolation.
3. Once the word was mastered in isolation, GA returned to the beginning of the sentence and attempted to produce the word correctly in sentence context. If the word was again produced in error, she returned to rehearsal of the word in isolation (see step 2, above) and again attempted the word in sentence context, until the entire sentence was produced correctly.

Treatment was focused on detection and correction of major speech sound errors or incomplete productions in multisyllabic words. Minor distortions (phonetic alterations that did not result in perception of a different phoneme, deletion/transposition/addition of a phoneme, or inability to produce a word in its entirety) were not targeted during training sessions because they did not disrupt intelligibility of speech to the same degree as major sound errors. Further, the participant could not be expected to reliably detect gradient phonetic changes to speech sounds. Sound prolongations and increased intersegment durations were also not targeted for treatment.

Treatment sessions were conducted by a licensed speech-language pathologist (M.H.) and lasted for one hour, once per week, for 12 weeks. During treatment sessions, one randomly-selected paragraph from the previous week's homework was read aloud in order to assess pronunciation of words therein. Treatment probe data were collected from these samples. Major sound errors were recorded on-line and total number of multisyllabic words with major sound errors was recorded for each session. In addition, all underlined words in the text (those that required rehearsal in isolation) were elicited in sentence context in order to provide additional accountability for homework practice. Words produced in error were practiced using steps 1–3 above. Subsequently, new text was trained using the above procedure (for a minimum of 30 minutes of oral reading). During each session, GA was asked to provide a spoken summary of the previous week's reading in order to encourage reading for comprehension at home. The purpose of sessions with the clinician was twofold: 1) to provide practice of multisyllabic words using the training procedure described above and 2) to check correct implementation of the training procedure in order to guarantee that home reading practice was conducted correctly.

Oral reading homework was completed at least five times per week for 30–45 minutes per day. The same text (typically 2–3 paragraphs) was reread several times per homework session, using steps 1–3 above, until the entire section was reliably produced without error. A book was selected by the clinician whose subject matter was of interest to the participant and which contained frequent multisyllabic words. The same text was used throughout. A representative sample of this text is included below:

> A shrinking minority of unreconstructed apologists for Leninism clung to the past; but from Berlin to Paris a new generation of Western progressives sought solace and example outside of Europe altogether, in the aspirations and upheavals of what was not yet called the "Third World'.
> –*Post War: A History of Europe since 1945* by Tony Judt

In the initial treatment session, it was observed that GA detected words produced in error (as defined above) during oral reading with 100% accuracy, and in subsequent treatment sessions, she maintained this ability to self-detect major speech errors with a very high degree of accuracy. On the rare occasion that GA produced an error and did not detect it, reading was stopped by the clinician and the word was rehearsed until correct production was achieved.

GA was encouraged to continue her home reading practice after weekly sessions (i.e., the active treatment phase) with the clinician were concluded. At each of the follow-up assessments, she confirmed that she had continued her reading practice at least 4–5 times per week since the conclusion of sessions with the clinician. We will refer to follow-up assessments as "post-treatment" time points because they were conducted after the conclusion of sessions with the clinician and because there was no longer any direct accountability for home practice. However, it should be noted that home practice did continue in the interim.

### 2.3. Assessment of treatment effects

Production of multisyllabic words during reading of novel texts was examined pre- and post-treatment and at follow-up assessments using the Gray Oral Reading Test-4 [30], which comprises passages of increasing difficulty and has two formats, which were used alternately. Each word was coded on-line as correctly produced on first attempt or incorrectly produced. Words that were not produced correctly were further sub-classified into those that were produced correctly after at least one repeated attempt and those that were not ultimately produced correctly (either after repeated attempts or not). Incorrect productions included incomplete attempts (i.e., partial productions) or those with speech sound errors (substitutions, additions, deletions or transpositions of sounds as well as distorted substitutions/additions). Minor sound distortions (gradient phonetic errors that did not result in perceived production of a different phoneme) were not counted as frank speech errors, but were coded. Samples were audiotaped and re-coded by the clinician after each session. For all reading probes, videotaped samples were also independently coded by another speech-language pathologist (S.T or M.M). Overall interrater reliability (percent agreement) for error coding was 93% and inconsistencies were resolved via consensus by reviewing videotaped samples. Speech errors were examined in connected speech via analysis of the WAB picture description using the same procedures.

The speech and language battery was repeated at post-treatment and follow-up sessions, using abbreviated versions of certain tests. Changes in performance on reading probes were examined using Wilcoxon signed rank tests. Performance on the Motor Speech Examination (MSE) was rated by a speech-language pathologist who did not see the patient for treatment (M.B.). The MSE was videotaped at each time point and the videos were reviewed and rated in a randomized order. Finally, we used a post-treatment survey, administered remotely via the internet, in order to gauge GA's perception of functional changes brought about by treatment.

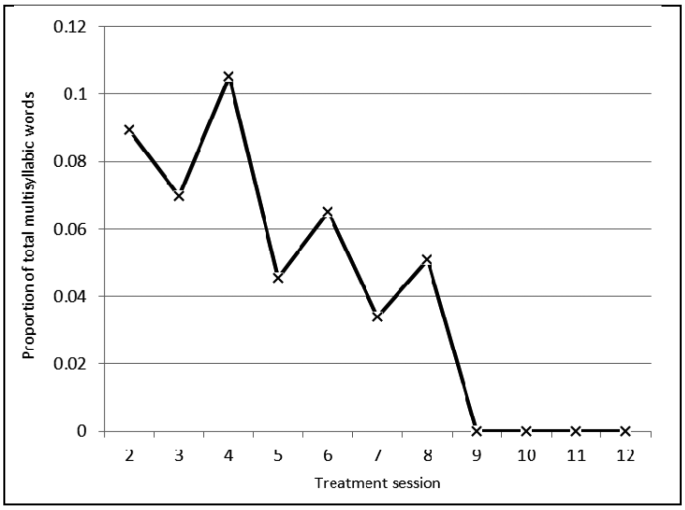

Fig. 2. Proportion of multisyllabic words produced with major speech errors in probes of trained homework text during treatment sessions.

## 3. Results

### 3.1. Multisyllabic word production during reading probes from trained homework text

Reading performance was probed in weekly sessions using a randomly-selected paragraph from text trained as homework the previous week. Performance on these probes (proportion of multisyllabic words with major speech sound errors) is depicted in Fig. 2. The figure shows that multisyllabic word production in trained text became more accurate and stable over the course of treatment and was error-free over the last four treatment sessions.

### 3.2. Multisyllabic word production during oral reading of untrained text

In order to assess whether our oral reading treatment could improve production of multisyllabic words during oral reading of difficult, untrained text and to examine the long-term effects of treatment, we administered the GORT-4 at post-treatment and follow-up assessments. Figure 3 depicts the proportion of multisyllabic words that were produced in error (excluding minor distortions) during reading probes. Wilcoxon signed rank tests (one-tailed) confirmed a significant reduction in speech errors on multisyllabic words at post treatment ($Z = -2.02$, $p = 0.02$), three months post-treatment ($Z = -2.02$, $p = 0.02$), six months post-treatment $Z = -2.02$, $p = 0.02$) and one year post-treatment ($Z = -1.75$, $p = 0.04$). When examining all word types together (single and multisyllable), very similar results were observed (post-treatment ($Z = -1.75$, $p = 0.04$), three months post treatment ($Z = -2.02$, $p = 0.02$), six months post-treatment treatment ($Z = -1.6$, $p = 0.05$), one year post-treatment treatment ($Z = -2.02$, $p = 0.02$)). These results confirm that oral reading treatment significantly improved production of multisyllabic words during text reading and that this pattern held when examining all word types combined. Figure 3 also shows the proportion of multisyllabic words produced in error that were self-corrected versus those that were not. There was a significant increase in the proportion of speech errors that were self-corrected at post treatment ($Z = -1.75$, $p = 0.04$) and at the three month follow-up assessment ($Z = -1.86$, $p = 0.03$). Six-month ($p = 0.17$) and one-year ($p = 0.24$) follow-up assessments were not significantly different from pre-treatment with regard to proportion of speech errors that were self-corrected.

Minor distortions (phonetic alterations that did not result in production of an incorrect speech sound or inability to produce a word in its entirety) were not targeted during training sessions; however, they were coded and analyzed for GORT-4 passages. Figure 4 shows the proportion of multisyllabic words that were produced with at least one minor distortion. The prevalence of minor distortions during oral reading of multisyllabic words did not change significantly from pre-treatment to post-treatment (two-tailed $p = 0.50$) or at the three or six month follow-up evaluations ($p = 0.69$ and $p = 0.14$, respectively). However, at one year post-treatment, there was a significant increase in the proportion of multisyllabic words produced with at

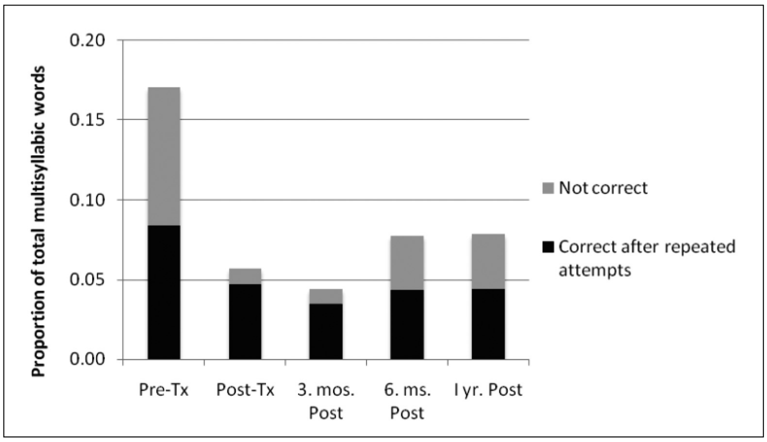

Fig. 3. Proportion of multisyllabic words with major speech errors on the Gray Oral Reading Test (GORT-4, passages 10–14).

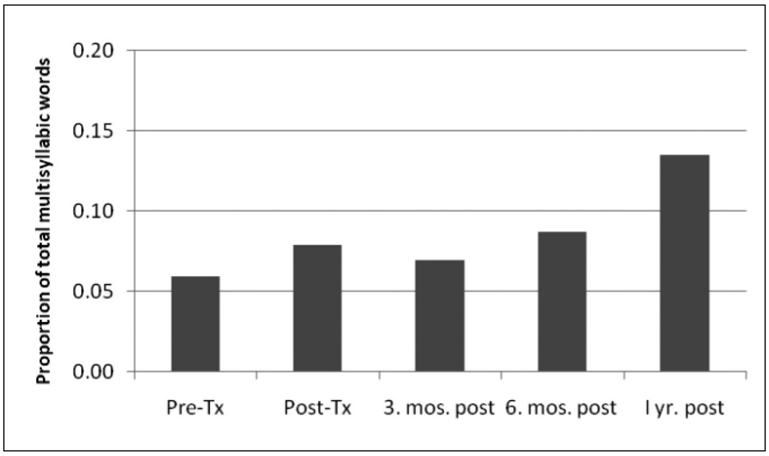

Fig. 4. Proportion of multisyllabic words with minor distortions (GORT-4).

least one minor distortion ($Z = -2.02, p = 0.04$). The same pattern was observed when all words were evaluated together (post-treatment, $p = 0.69$; three months post, $p = 0.69$; six months post, $p = 0.23$; one year post-treatment ($Z = -2.02$, $p = 0.04$)). These findings indicate that the rate of occurrence of minor distortions did not increase or decrease as a result of treatment and that these behaviors remained stable until one year post-treatment, at which point their frequency increased. Figure 5 depicts reading rate in words per minute, averaged across the five GORT passages at each time point. Reading of novel text showed a gradual slowing over time. Reading rate was not significantly slower at post-treatment (one-tailed $p = 0.25$) or three months post-treatment ($p = 0.11$), but was significantly slower at six-month ($Z = -2.02$, $p = 0.02$), and one year ($Z = -1.76$, $p = 0.04$) follow-up assessments.

In order to examine generalization of treatment effects to untrained tasks and contexts, GA's speech production was assessed during repeated production of mono- and trisyllabic words and during sentence repetition (Table 1). Repetition of monosyllabic words (x5) and short sentences remained stable across time points. Tri-syllabic word repetition improved considerably from pre- to post-treatment, with maintenance at three months, six months, and one year post-treatment. Repetition of long sentences was improved post-treatment, with some degree of maintenance at three and six months post-treatment. GA's speech production was also examined during connected speech in a picture description task from the Western Aphasia Battery (Fig. 6). The frequency of major speech sound errors and minor distortions in connected speech did not change considerably from pre-treatment to any of the post-treatment time points.

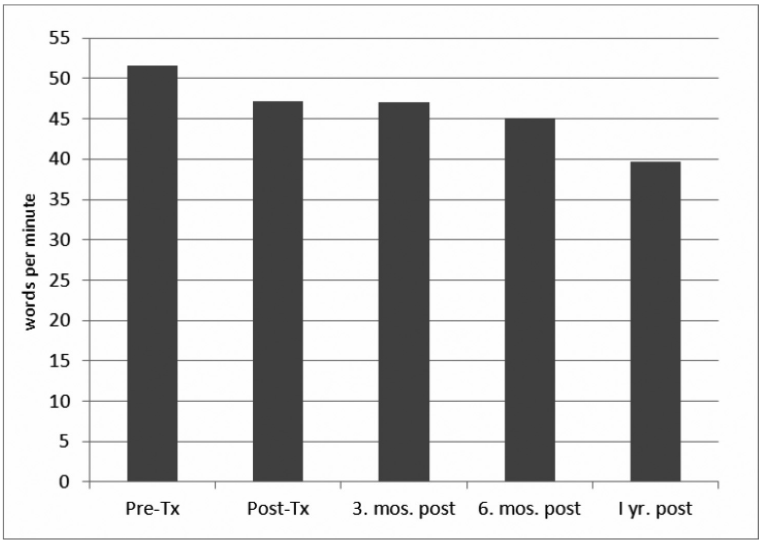

Fig. 5. Reading Rate for GORT-4 passages.

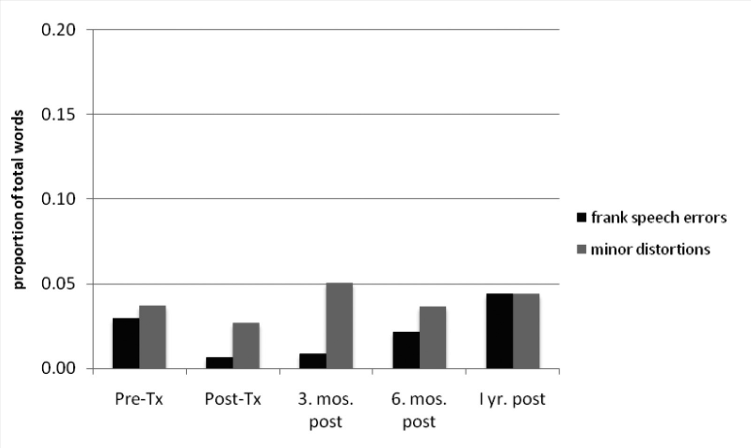

Fig. 6. Speech errors during picture description from the WAB.

### 3.3. *Post-treatment and follow-up speech and language evaluations*

Scores on the WAB remained relatively constant across treatment and follow-up assessments up to the six month follow-up, as reflected by little change in the Aphasia Quotient (Table 1). At the one year follow-up assessment, WAB AQ dropped nearly seven points. This change was due to a reduction in the fluency rating brought about by impaired speech production. AOS rating on the Motor Speech Evaluation did not change from pre-to post-treatment. At three months post-treatment, AOS severity was noted to have worsened. Changes in voice quality (harshness and breathiness) and decreased pitch and loudness were also observed. At one year post-treatment, AOS severity was again noted to have worsened. Changes in AOS severity at 3 months and one year were largely due to an increase in what we have termed "minor distortions" as well as a slower rate of speech, increased frequency of pauses within and between words, and further reduction in prosody. Syntactic and semantic assessments and single-word reading/spelling showed little change over time.

### 3.4. *Post-treatment survey*

Table 2 shows results of a post-treatment survey examining GA's self-ratings of treatment effects. GA in-

Table 2
GA's self-ratings of treatment effects

| Since you began oral reading treatment, how would you rate your | a lot worse (1) | worse (2) | some-what worse (3) | un-changed (4) | some-what better (5) | better (6) | a lot better (7) |
|---|---|---|---|---|---|---|---|
| −Reading fluency (ability to read smoothly and without errors) | | | | | | X | |
| −Speaking fluency (ability to speak smoothly and without errors) | | | | | X | | |
| −Comfort level while reading aloud | | | | | | | X |
| −Comfort level while speaking | | | | | X | | |
| −Reading comprehension (ability to understand written passages) | | | | X | | | |
| −Word production (ability to produce single words with several syllables) | | | | | | X | |
| −Sentence production (ability to speak in complete sentences without errors) | | | | | | X | |
| −Ability to detect any errors in your speech | | | | X | | | |
| −Ability to correct your own speech errors when they occur | | | | X | | | |
| −Ability to assign proper stress to words when reading or speaking | | | | | | X | |
| −Confidence in communication with your primary communication partner (e.g., spouse) | | | | X | | | |
| −Confidence in communication with familiar people | | | | | | X | |
| −Confidence in communication with unfamiliar people | | | | | X | | |
| −Frustration level during communication exchanges with primary communication partner | | | | | X | | |
| −Frustration level during communication exchanges with familiar people | | | | | | X | |
| −Frustration level during communication exchanges with unfamiliar people | | | | | X | | |

dicated that she perceived improved performance and comfort level in reading aloud and speaking (fluency), as well as improvements in multisyllabic word production, sentence production, and stress assignment in words post-treatment. She also indicated improved confidence and reduced frustration in communication with both familiar and unfamiliar people as a result of treatment.

## 4. Discussion

Individuals with the nonfluent variant of PPA often face a slow decline in motor programming ability for speech, or AOS, in the relative absence of other linguistic and cognitive deficits [16,23]. These patients ultimately become mute and reliant on augmentative and alternative communication (AAC) strategies/devices. Whereas AAC approaches provide an important substitute for or complement to spoken communication in many individuals, the loss of spoken language is devastating and debilitating and patients frequently express a desire for behavioral treatment options to help manage motor speech deficits.

Individuals with PPA may benefit from strategies to improve speech production when their impairments are still mild and, potentially, slow the inevitable progression of their difficulties. Despite the need for rehabilitation strategies addressing the motor speech deficits observed in nonfluent variant PPA, there have been virtually no investigations of such treatments that we are aware of to-date. Attempts to rehabilitate AOS in stroke patients, while promising, are often not appropriate in patients with mild AOS due to progressive disease. These treatments are typically designed to address severe motor speech planning deficits, with single sound, syllable, or monosyllabic word targets that are not appropriate for mild cases.

We attempted to address the need for treatment research in this area by implementing a novel treatment approach with an individual with mild AOS associated with nonfluent PPA. The treatment was designed to provide a context within which to rehearse self-detection and correction of speech sound errors due to AOS, with the specific goal of improving multisyllabic word production during oral reading. We sought to determine whether structured oral reading practice can improve production of multisyllabic words during text reading, whether treatment effects generalize to untrained behaviors, and also to examine the long-term effects of treatment.

Over the course of this intervention, GA showed a decrease in major speech errors on multisyllabic words in trained text, with error-free reading of these words over the last few treatment sessions. She also showed a decrease in major speech errors on multisyllabic words (and all words) when reading difficult untrained text (GORT-4 passages 10–14) as well as an increase in successful self-corrections post-treatment. This improvement was maintained up to 1 year following the completion of treatment with the clinician, with continued home practice. The frequency of speech sounds with minor distortions did not change from pre- to post-treatment, but did remain stable until at least six months after treatment with the clinician was completed. With regard to generalization, we observed improved speech production during repetition of long sentences at post-treatment and improvement on repeated production of trisyllabic words up to one year post-treatment, suggesting that treatment effects generalized to untrained tasks.

In addition, we observed little change in the frequency of major speech sound errors during connected speech probes up to one year post-treatment, suggesting that treatment may have helped to stabilize speech production in non-reading contexts. However, an increase in phonetic distortions on consonants and vowels, slowed speech rate, and an increase in the number of pauses within and between words resulted in a greater AOS severity rating on the MSE, a test that is designed to elicit motor speech errors in AOS and dysarthria by taxing the speech production system with a variety of tasks. This worsening of the AOS severity rating reflects a continuing decline in certain aspects of motor programming for speech that was not captured by oral reading and connected speech probes. For example, diadochokinetic productions were increasingly slow and irregular over time. In addition, the MSE rating is a subjective measure that likely takes into account the overall effortfulness of speech production and visually observable behaviors, such as groping of the articulators, which were not reflected in our treatment outcome measures.

In addition to our observations that speech error rates declined as a result of treatment, GA indicated that she perceived improvement in her own speaking and reading, as well as increased confidence and decreased frustration when communicating with others. She also reported that friends and family who were unaware of her participation in this study indicated that her speech intelligibility was improved after treatment.

Oral reading of text provides a context within which to rehearse production of multisyllabic words in connected speech. Treatment techniques for AOS have incorporated written word forms, but no previous treatment paradigm has focused on use of the written word as a primary cue for speech production. Our method, wherein GA broke the written word down into constituent syllables and produced the word syllable-by-syllable, may have provided visual cues via orthography that helped to guide implementation of the correct speech motor program. Short words are typically produced with less difficulty in individuals with AOS, likely because of reduced complexity of motor programming. By breaking multisyllabic words into constituent syllables, the motor programming demands are reduced to syllable-sized chunks. Application of this technique in the context of reading aloud enables the visual word form to serve as a guide to correct production of each sound and syllable in the proper sequence.

It is our conclusion that changes in GA's oral reading and speech performance reflected improvements in motor planning for multisyllabic words. However, we acknowledge that our classification scheme for speech sound errors in this study assumed that all errors were motor, rather than phonological, in nature, even when sounds were not produced in a distorted manner. Major sound errors, which were the object of treatment, were defined as distorted or non-distorted productions that resulted in perception of a substituted, deleted, transposed, or added phoneme or in an incompletely produced target. We cannot rule out the possibility that some portion of these errors arose at a phonological level. However, GA's overall pattern of speech impairment was highly consistent with AOS, she was classified as non-aphasic by the WAB (AQ of 93.8 or higher), and she performed within the normal range on measures of naming, reading, and writing, all of which reflect a relative sparing of phonological processing. Therefore, we are confident that the majority of errors targeted in treatment and quantified by our outcome measures arose at the level of motor planning and that this is the level at which change was brought about by treatment.

Our preliminary findings in this case suggest that structured oral reading treatment for multisyllabic words may hold promise as a feasible, cost-effective, and efficient means of delivering therapy for patients with nonfluent variant PPA. First, it is simple to implement and can be tailored to any difficulty level, from very complex text, as was used with GA, to simple passages conveying personal information that may serve as material for script training in more severely apraxic individuals. Our participant, GA, was very accurate at self-detection of major speech sound errors and failed attempts at word production. This skill is critical to the implementation of this procedure outside the clinic.

Once it is ascertained that the participant can reliably detect speech sound errors, however, it is possible to complement treatment sessions with the clinician with homework practice, allowing the individual to receive therapeutic stimulation daily. It is quite likely that GA's maintenance of treatment effects after cessation of sessions with the clinician was due to her continued oral reading practice at home.

Finally, the treatment has the potential to stimulate and even improve grammatical processing in agrammatic patients, as has been shown with other oral reading treatments in aphasia resulting from stroke [20]. This is important given that many individuals with nonfluent PPA will ultimately develop impairments of syntactic comprehension and production with disease progression. Thus, oral reading treatment may prove beneficial for both motor speech and, ultimately, syntactic impairments observed in nonfluent variant PPA. Because GA was still near ceiling on measures of syntactic processing post-treatment, the latter will require systematic examination in future patients.

We have presented a novel approach to treatment of motor speech impairment in nonfluent variant PPA. This structured oral reading treatment, designed to facilitate self-detection and correction of speech errors on multisyllabic words, resulted in a reduction of speech errors on trained and untrained tasks and behaviors that was maintained for one year after the end of treatment with the clinician. This study provides an initial account of the utility of this type of treatment in mild AOS, which will need to be explored further and replicated with a larger group of individuals.

## Acknowledgements

We wish to thank our research participant for all of her hard work and dedication. This work was supported by the NIH (NIDCD F32DC010945, NIA P01 AG019724, and NINDS R01 NS050915).

## References

[1] M.L. Gorno-Tempini, A.E. Hillis, S. Weintraub, A. Kertesz, M. Mendez, S.F. Cappa et al., Classification of primary progressive aphasia and its variants, *Neurology* **76**(11) (2011), 1006–1014.

[2] M.L. Gorno-Tempini, N.F. Dronkers, K.P. Rankin, J.M. Ogar, L. Phengrasamy, H.J. Rosen et al., Cognition and anatomy in three variants of primary progressive aphasia, *Ann Neurol* **55**(3) (2004), 335–346.

[3] M. Mesulam, A. Wicklund, N. Johnson, E. Rogalski, G.C. Leger, A. Rademaker et al., Alzheimer and frontotemporal pathology in subsets of primary progressive aphasia, *Ann Neurol* **63**(6) (2008), 709–719.

[4] K.A. Josephs, J.R. Duffy, E.A. Strand, J.L. Whitwell, K.F. Layton, J.E. Parisi et al., Clinicopathological and imaging correlates of progressive aphasia and apraxia of speech, *Brain* **129** (2006), 1385–1398.

[5] E. Ratnavalli, C. Brayne, K. Dawson and J.R. Hodges, The prevalence of frontotemporal dementia, *Neurology* **58**(11) (2002), 1615–1621.

[6] D. Knopman, R. Petersen, S. Edland, R. Cha and W. Rocca, The incidence of frontotemporal lobar degeneration in Rochester, Minnesota, 1990 through 1994, *Neurology* **62**(3) (2004), 506–508.

[7] C. Taylor, R.M. Kingma, K. Croot and L. Nickels, Speech pathology services for primary progressive aphasia: Exploring an emerging area of practice, *Aphasiology* **23**(2) (2009), 161–174.

[8] M. Henry, Treatment for Progressive Impairments of Language, *Perspectives on Neurophysiology and Neurogenic Speech and Language Disorders* **20**(1) (2010), 13–20.

[9] K. Croot, L. Nickels, F. Laurence and M. Manning, Impairment-and activity/participation-directed interventions in progressive language impairment: Clinical and theoretical issues, *Aphasiology* **23**(2) (2008), 125–160.

[10] S.L. Schneider, C.K. Thompson and B. Luring, Effects of verbal plus gestural matrix training on sentence production in a patient with primary progressive aphasia, *Aphasiology* **10**(3) (1996), 297–317.

[11] B. Rapp and B. Glucroft, The benefits and protective effects of behavioural treatment for dysgraphia in a case of primary progressive aphasia, *Aphasiology* **23**(2) (2009), 236–265.

[12] R. Jokel, E. Rochon and C. Leonard, Treating anomia in semantic dementia: improvement, maintenance, or both? *Neuropsychol Rehabil* **16**(3) (2006), 241–256.

[13] S.L. Schneider and C.K. Thompson, Verb production in agrammatic aphasia: The influence of semantic class and argument structure properties on generalisation, *Aphasiology* **17**(3) (2003), 213–241.

[14] M. Louis, R. Espesser, V. Rey, V. Daffaure, A.D. Cristo and M. Habib, Intensive training of phonological skills in progressive aphasia: a model of brain plasticity in neurodegenerative disease, *Brain Cogn* **46**(1–2) (2001), 197–201.

[15] R. Jokel, J. Cupit, E. Rochon and C. Leonard, Relearning lost vocabulary in nonfluent progressive aphasia with MossTalk Words®, *Aphasiology* **23**(2) (2009), 175–191.

[16] K.A. Josephs, J.R. Duffy, E.A. Strand, M.M. Machulda, M.L. Senjem, A.V. Master et al., Characterizing a neurodegenerative syndrome: primary progressive apraxia of speech. Brain in press.

[17] J. Wambaugh, J. Duffy, M. McNeil, D. Robin and M. Rogers, Treatment guidelines for acquired apraxia of speech: A synthesis and evaluation of the evidence, *Journal of Medical Speech Language Pathology* **14**(2) (2006).

[18] J.R. Duffy, Motor speech disorders. St. Louis: Mosby, Incorporated, 1995.

[19] L. Cherney, Efficacy of oral reading in the treatment of two patients with chronic Broca's aphasia, *Topics in Stroke Rehabilitation* **2**(1) (1995), 57–67.

[20] S. Orjada and P. Beeson, Concurrent treatment for reading and spelling in aphasia, *Aphasiology* **3**(5) (19 2005), 341–351.

[21] M.M. Mesulam, Primary progressive aphasia, *Ann Neurol* **49**(4) (2001), 425–432.

[22] R.T. Wertz, L.L. LaPointe and J.C. Rosenbek, Apraxia of speech in adults: The disorder and its management, New York: Grune and Stratton; 1984.

[23] J.M. Ogar, N.F. Dronkers, S.M. Brambati, B.L. Miller and M.L. Gorno-Tempini, Progressive nonfluent aphasia and its characteristic motor speech deficits, *Alzheimer Disease and Associated Disorders* **21**(4) (2007), S23–S30.

[24] S.M. Wilson, N.F. Dronkers, J.M. Ogar, J. Jang, M.E. Growdon, F. Agosta et al., Neural Correlates of Syntactic Processing in the Nonfluent Variant of Primary Progressive Aphasia, *The Journal of Neuroscience* **30**(50) (2010), 16845–16854.

[25] D. Howard and K. Patterson, Pyramids and palm trees: A test of semantic access from pictures and words, Bury St.Edmunds, UK: Thames Valley Test Company 1992.

[26] E. Kaplan, H. Goodglass and S. Weintraub, Boston Naming Test. Philadelphia: Lippincott, Williams and Wilkins, 2001.

[27] P.M. Beeson, K. Rising, E.S. Kim and S.Z. Rapcsak, A treatment sequence for phonological alexia/agraphia, *Journal of Speech, Language, and Hearing Research* **53**(2) (2010), 450–468.

[28] J.V. Baldo, D.P. Wilkins, J. Ogar, S. Willock and N.F. Dronkers, Role of the precentral gyrus of the insula in complex articulation, *Cortex* **47**(7) (2010), 800–807.

[29] K.A. Bayles, C.K. Tomoeda and J.A. Rein, Phrase repetition in Alzheimer's disease: effect of meaning and length, *Brain Lang* **54**(2) (1996), 246–261.

[30] J.L. Wiederholt and B.R. Bryant, Gray oral reading tests-(GORT-4). Austin, TX: Pro-Ed 2001.
